# The Impact of Shared Information Presentation Time on Users’ Privacy-Regulation Behavior in the Context of Vertical Privacy: A Moderated Mediation Model

**DOI:** 10.3390/bs13090706

**Published:** 2023-08-25

**Authors:** Lei Zhuang, Rui Sun, Lijun Chen, Wenlong Tang

**Affiliations:** 1 School of Business Administration, Huaqiao University, Quanzhou 362000, China; 2 Oriental Business Management Research Center, Huaqiao University, Quanzhou 362000, China; 3 School of Business Administration, Jimei University, Xiamen 362000, China

**Keywords:** digital platforms, recommendation algorithms, survey method, information sharing, presentation time, privacy-regulation behavior, online vigilance

## Abstract

Combining data-sharing models and algorithm technologies has led to new data flow structures and usage patterns. In this context, the presentation time of shared low-sensitivity information across platforms has become a crucial factor that affects user perception and privacy-regulation behavior. However, previous studies have not conducted an in-depth exploration of this issue. Based on privacy process theory, this study discusses the impact and potential mechanism of the presentation time (immediate or delayed) of shared low-sensitivity information across platforms on privacy-regulation behavior. Through a pre-study and two online survey experimental studies, which included 379 participants in total, we verified that the immediate information presentation time has a significantly higher impact on online vigilance and privacy-regulation behavior than the delayed condition, β_direct_ = 0.5960, 95% CI 0.2402 to 0.9518; β_indirect_ = 0.1765, 95% CI 0.0326 to 0.3397, and users’ perceived control as the moderating role influences online vigilance and privacy-regulation behaviors (preventive or corrective), β_preventive_ = −0.0562, 95% CI −0.1435 to −0.0063; β_corrective_ = −0.0581, 95% CI −0.1402 to −0.0065. Based on these results, we suggest that the presentation time of using shared low-sensitivity information across platforms should be concerned by companies’ recommendation algorithms to reduce users’ negative perceptions and privacy behaviors and improve user experience.

## 1. Introduction

Through multimodal communication [1], the combination of data-sharing models and algorithm technologies has led to novel data flow structures and usage patterns, which have altered users’ perceptions and reactions toward the use of personal data on mobile platforms [2]. For example, several mainstream applications (apps), such as Facebook, Twitter, and WeChat, have an increasing number of related parties and authorized partners with whom they share certain low-sensitivity information through the third-party service agreements that users have to authorize before using the service [3,4,5,6]. Concurrently, real-time recommendations with faster response capabilities [7,8,9] are widely adopted in these apps. The combination of platforms’ data stream sharing structure and real-time algorithms has improved the accuracy and responsiveness of apps’ personalized services [10]. However, scholars have cautioned that the combination of information flow and algorithm will produce qualitative changes in users’ perception of how data sharing affects their privacy; thus, the low-sensitivity information, previously regarded as less impactful on users’ privacy behaviors [2], could become an important influencing factor in this context [1,11]. Additionally, the massive usage of users’ low-sensitivity information in recent years has increased privacy-regulation behaviors, such as diving, closing personalized services, or directly refusing to use apps [12,13,14]. Many countries have introduced laws to empower users to refuse personalized services in the recent past, thus bringing immense challenges to platforms in terms of maintaining users’ activity and accessing personal data. Therefore, further in-depth study on the impact of sharing low-sensitivity information across platforms in novel algorithm contexts on users’ perception and privacy behavior is required.

Previous studies on the impact of shared information on user privacy behavior have mainly focused on the relationship between social media information sharing among user groups and user privacy disclosure behavior, privacy concerns, and authorization behavior [11,15]. Research on the impact of the collection and use of personal information by personalized algorithm technology on users’ privacy behavior and tendencies mainly focused on information-collection methods (hidden or open) [16,17], information-storage programs [18], information transparency [19,20], the relevance of advertising information [21], technology insecurity [22], illegal use of sensitive data [23], platform commitment [24,25], and other factors.

However, there are two problems with these prior studies. First, several previous investigations of personal privacy behavior caused by information sharing have focused on individuals’ active privacy behavior against potential threats to the users’ privacy in the horizontal privacy context. However, less attention has been paid to privacy behavior after users’ passive consent to share their personal information with the third-party platform, referred to as the vertical privacy context [2]. The loss of sensitive information not authorized by the users can be measured in the horizontal privacy context [23] by calculating the severity and probability of risk to explain users’ privacy behavior [22,26,27,28,29]. Nevertheless, in the vertical privacy context where information has been authorized by users at the beginning, the users cannot fully understand the underlying technology and rules of information sharing flow across platforms; thus, potential threat during the process of sharing low-sensitivity information among platforms for users is difficult to calculate using the loss and the probability of occurrence in the Privacy Calculus theory [1].

Second, theories such as Communication Privacy Management (CPM) suggest that the unsuitable timing of the release of unauthorized sensitive information to third parties can lead to the cessation of the privacy boundary from the original information discloser [30,31,32]. However, these theories failed to note how people perceived the low-sensitivity information already authorized across platforms and the strategies they would adopt in response. The Privacy Process Model theory (PPM), which was developed based on CPM theory, posits the necessity to analyze the characteristics of specific new technologies to discuss users’ perceptions and behavior and provides a theoretical framework to explain why people continue to disclose their information when perceived privacy risks exist [1]. 

However, so far, the PPM theory has not been applied in an empirical study focusing on users’ responses to the characteristics of specific new technologies, and the theory does not consider the collective privacy regulatory behavior involving information collection and usage by third-party apps and service suppliers [31,32]. Therefore, analysis of the impact of the presentation time of the sharing of low-sensitivity information on users’ perception of online vigilance and privacy-regulation behavior within the framework of the PPM theory would extend the theory’s application in the combined context of across-platform data-sharing models and real-time algorithm technology.

Altogether, this study investigated the effect of presentation time of shared low-sensitivity information across platforms and the boundary conditions of perceived control on users’ perception and privacy-regulation behaviors within the framework of the PPM theory. This study explored the collective privacy relationship between the users, the platform they used, and the third-party platform in the process of sharing users’ low-sensitivity information across platforms. This study expanded on users’ perceptions and privacy-regulation behaviors in regard to algorithm technology in the big data context. These findings will help to reduce users’ negative perceptions and privacy behaviors and inform digital platforms on how to improve real-time algorithms and user loyalty. 

The rest of this study is arranged in the following manner: in the next section, the background of the research is explained based on the existing literature, followed by the research methods, results, discussion, and conclusions, along with theoretical and practical implications and limitations, which include potential directions for follow-up studies.

## 2. Literature Review and Research Hypothesis

### 2.1. Vertical Privacy

With the technological development of networks and big data technologies, users face a new economy in which personal information is a commodity. A platform can collect as much user information as possible and then summarize and analyze it to infer the user’s interests and purchase intentions. In this context, scholars have gradually started to distinguish vertical and horizontal privacy. Horizontal privacy refers to the threat from other users that results in the malign and uncontrolled spread of personal information on social media. Vertical privacy refers to the threat from the network platforms and institutions which collect and use a large amount of user information. At the vertical level, a platform starts gathering personal information as authorized through user registration, the agreement to the “service terms” and “privacy statement” of the online platform, and the agreement to authorize third-party application services. The platform can continuously track and collect information on users’ behavior and activity and even share or transfer collected low-sensitivity personal information with third-party applications.

Scholars point out that further study on vertical privacy is particularly important for the following reasons. First, vertical privacy threats originate from the progress of online service technology, both software and hardware, and have become increasingly prominent with the development of big data technology. Furthermore, different technologies vary in terms of the information flow characteristics of the unprecedented amount of collected users’ information, thus making it difficult for users to control vertical privacy risks. Second, compared to the horizontal level of self-disclosure, the vertical privacy issue is more difficult to recognize, leading to delayed awareness or even total ignorance of the existence of this concept by users. Previous studies showed that while users have taken various measures to protect their horizontal privacy, they remain largely uncertain about how to protect their vertical privacy. However, if people can perceive or understand the vertical characteristics of the collected and analyzed information, they will exhibit behavioral responses. For example, when participants became aware of how often some of their sensitive data are accessed by other apps, 95% of them reassessed their privacy settings, and 58% of the participants further restricted some of them [33]. Making people aware of the inferences that can be made based on the data they provide can make them more concerned about releasing this information and more cautious about posting it online [34]. Therefore, it is necessary to conduct an in-depth study on the impact of technical features on user privacy behavior in vertical privacy scenarios during the actual process of online communication services.

### 2.2. Privacy-Regulation Behavior

Privacy-regulation behavior refers to specific actions taken by people to adjust the privacy level they are facing to maintain social interactions while avoiding risks—in other words, individuals need to disclose themselves appropriately while achieving their comfort level of privacy protection [1]. Regulation of personal space and physical contact (also referred to as haptic and spatial privacy regulation), time, physical appearance, etc., can help to obtain the desired level of social interaction [1]. In the network environment, individuals’ information regulation behavior includes controlling and managing their own information by choosing which data to disclose or not (i.e., information hiding) [32]. 

Individual privacy-regulation behavior online can be categorized into preventive and corrective behavior. Preventive behaviors were identified, such as using pseudonyms and false information, creating a group friend list or adjusting privacy settings to limit the visibility of personal information, using different accounts to show different content, using anti-tracking software, and adopting a secret language [31,35]. Corrective behaviors include deleting published or disclosed content, choosing to quit some online activities, turning off personalized recommendation and advertising options, changing social platforms, and blacklisting or deleting friends [2,36].

Substantial literature shows that people have various privacy-regulation behaviors when they are aware of privacy threats online; however, most existing research focuses on horizontal privacy threats and less on the privacy-regulation behaviors towards vertical privacy threats caused by data sharing across multiple platforms [1].

### 2.3. Privacy Process Model Theory

PPM provides a theoretical framework to model how people perceive and respond in different privacy situations and explains why people still choose to disclose their information even when they perceive privacy risks. PPM is based on the assumption that individuals evaluate whether they have reached the desired privacy level through their subjective perception of the objective privacy situation and then adjust the level of self-disclosure to change the environment (i.e., privacy regulation) [37]. By improving on the CPM theory, which posits that people lose their privacy through self-disclosure, PPM has the following key features: (1) it regards privacy as the degree of separation from others, (2) it distinguishes between objective and subjective privacy perception, and (3) it integrates the concept of privacy regulation and self-disclosure [1]. The self-disclosure adjustment behavior of individuals is regarded as a useful function to adjust the privacy level in PPM [37]. For example, deliberately altering the options of the website cookie banner can manipulate the outcome of users’ privacy decisions and “nudge” users into sharing their data [38]. The PPM theory includes four main elements: privacy context, privacy perception, privacy-regulation behavior, and privacy controllability [37]. However, PPM is still ambiguous about the specific characteristics of privacy situations and ignores the collective privacy management process in multimodal communication [1]. Thus, further in-depth study on the impact of specific characteristics on privacy-regulation behavior is needed.

### 2.4. Information Presentation Time and Privacy-Regulation Behavior

The shared information presentation time across platforms refers to when user-authorized data are shared and transferred to the current platform and emerge as the recommended content. It can also refer to the time that recommended content based on cross-platform shared data updated by the recommendation algorithms is presented to the users. In a real-time or streaming recommendation situation [7,8,9], the data’s update and presentation time varies depending on the recommendation algorithms, which are typically based on the combination of two strategies that generate the updated recommendation results: (1) regular collection of user data for offline modeling and updating, or (2) online real-time update based on the user’s behavior in the recent seconds [7,8,9,39]. The latter is quite popular, with several mainstream apps, including Tiktok and Alibaba, adopting real-time recommendation algorithms.

In previous studies, scholars found that the presentation time of personalized recommendation had a significant impact on user behavior (e.g., click behavior), and some studies found that the presentation time (immediate or delayed) of personalized recommendation influenced the recommendation effect [40,41]. Burgoon et al. studied the impact of time on privacy behavior in the non-network context and realized that people could achieve privacy by time while allowing the territory to overlap in space, for example, people avoiding crowded low-privacy environments by taking public transport during off-peak hours [42] or delaying a reply to privacy violation dialogue to isolate the immediate privacy risks [42]. On the internet, the privacy regulation of users also involves time-level management behaviors. For example, due to the persistence of data storage technologies on social media, users delete or withdraw some content that could cause a risk in the future [41]. Tufekci also noted that current privacy decisions could cause problems in the future and suggested that further studies on the “time” boundary of privacy are required [43]. Petronio’s CPM theory defines the time when individuals’ sensitive information is disclosed from the second party to the third parties as the factor of linkage when the binary privacy information boundary is converted into collective multiple boundaries. Individuals evaluate the appropriateness of disclosure according to the transferring time to decide on the opening or closing of the private information boundary; if the transferring time of sensitive information is too short, then individuals are more likely to choose closed information boundaries [44]. However, studies based on the CPM theory mostly focus on sensitive information and thus cannot fully explain the perception of low-sensitivity information and privacy-regulation behavior caused by the transferring time of such information.

Therefore, in the vertical privacy context, which combines sharing technologies across platforms and real-time algorithms, we believe that the presentation time characteristic is the main factor that leads to users’ regulation behaviors. Users adjust the privacy-regulation behavior according to the transferring and presenting time among the multiple boundaries of information. Compared with the delayed condition, an immediate presentation time would lead to a higher privacy-regulation behavior. Consequently, we propose:

**H1:**  *The presentation time of shared information among platforms negatively affects users’ privacy-regulation behavior.*

### 2.5. Online Vigilance

The concept of online vigilance was proposed by Klimmt et al. to explain the perception of blurred boundaries and work–life conflicts among social media users caused by the “always online” high responsiveness of the immediate communication technology [45]. For social media with job-like attributes, users’ workmates need to reply, follow, release, comment, or provide likes to the users’ updated information, which means that all users’ peers may be able to monitor them virtually [45]. The more immediate the other people’s responses are, the higher the perception of online vigilance is [46,47]. From the information processing perspective, different timing leads to different perceptions. For example, the accessibility–diagnosability theory posits that the ease of access to information from one’s memory is related to the likelihood of using this information to diagnose a situation the individual is facing, and that the time interval since the individual last activated the information cognition leads to different perception to the situation [48]. Information processing theory stipulates that a closer time interval among the events leads to their higher cognitive relevance to an individual and a higher perception of the relevance of the events [49,50]. Therefore, the closer the time of the potential threat, the higher the likelihood that it is perceived as more relevant.

Although it is difficult to calculate and measure the authorized low-sensitivity information from the dimensions of perceived severity and probability, the real-time recommendation algorithms make for the high response speed of user information sharing among platforms, which leads to users’ online vigilance. The shorter the information-sharing presentation time, the higher the online vigilance of the users. An immediate information presentation time causes higher user online vigilance than a delayed situation. Consequently, we propose:

**H2:**  *The shared information presentation time negatively affects users’ online vigilance.*

Bossi et al. found that online vigilance generated by the high responsiveness of working social media leads to burnout or emotional exhaustion, resulting in social media avoidance [47,51]. An increase in online vigilance leads to more serious avoidance behavior [52]. Liang and other scholars noted that potential network security threats caused by technology improvements generate a sense of urgency among users—the stronger the threat perception is, the more likely they are to take protective actions, such as closing cookies or avoiding a sense of urgency through post-corrective actions [28,29]. Therefore, we posit that a higher online vigilance awareness leads to a higher privacy-regulation behavior:

**H3:**  *Online vigilance positively affects users’ privacy-regulation behavior.*

### 2.6. Perceived Control

Perceived control related to privacy behavior refers to how much the users feel in control of their personal privacy information [53]. In personalized services, Tucke found that if websites’ privacy settings offer options such as “opt out” that can be used to enhance consumers’ perceived control of personal information, consumers’ click rate on personalized ads generated by using their personal data is improved [54]. Song et al. noted that when consumers feel they have more control over personal information, the impact of personalized privacy risks on the consumers’ privacy behavior decreases, even when they perceive privacy threats [55]. Brandimarte et al. manipulated participants’ perceived control of information in the experiment and found that participants who clearly perceived that they could control the release and access of personal information would disclose more personal information—a sense of more control makes users more inclined to ignore privacy risks [56].

Compensatory control theory notes that acquiring and maintaining a high sense of personal control is a basic human need [57]. When people believe their control is threatened, they are motivated to restore it [58,59] by adopting different following strategies. First, a person’s sense of control is enhanced by solving problems. Thus, when people feel threatened by losing control, they become motivated to solve their current problems [57] using their own ability and resources to restore their sense of control. Moreover, a lack of personal control leads to an increased desire to establish order and structure to draw tangible or intangible boundaries that can increase individuals’ sense of control in response to threats [60]. In addition, the lack of sense of control makes it easier to believe or recall exaggerated opinions and news, such as conspiracy theories and negative news, and users tend to seek an explanation for the chaotic outside world to make it feel more understandable and controllable [58,61]. Therefore, during the collection and use of cross-platform low-sensitivity information, users with low perceived control are more likely to recall news and opinions that report on privacy violation issues, stimulate their privacy awareness, and exhibit a desire to establish invisible psychological boundaries. All these feelings generate online vigilance and privacy-regulation behavior to restore the sense of control.

Therefore, this study assumes that a low perception of control strengthens the relationship between online vigilance and corrective vs. preventive behaviors and thus enhances corrective vs. preventive behaviors. A low perception of control strengthens the relationship between the information presentation time and both corrective and preventive behaviors, thereby enhancing corrective vs. preventive behaviors in advance. Consequently, we propose:

**H4:**  *Perceived control moderates the relations between online vigilance and preventive behavior and between the information presentation time and preventive behavior.*

**H5:**  *Perceived control moderates the relations between online vigilance and corrective behavior and between the information presentation time and corrective behavior.*

### 2.7. Research Model

**Figure 1:**
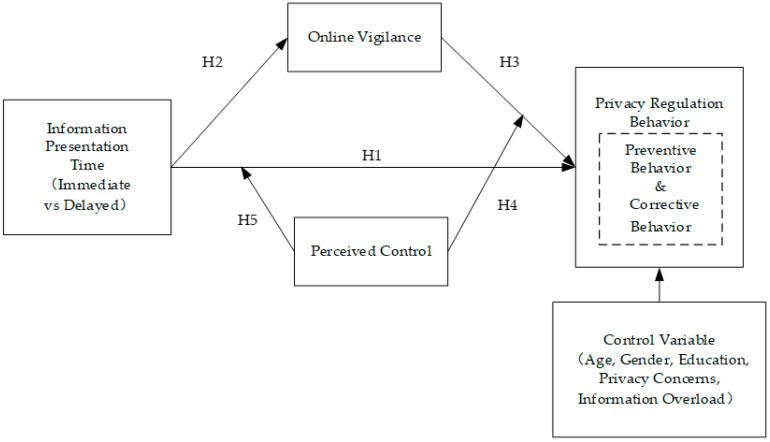
The conceptual model.

The conceptual model of this study is shown in Figure 1. The independent variable is the presentation time of information shared between platforms; the dependent variable is privacy-regulation behavior (including preventive behavior and corrective behavior); the mediating variable is online vigilance; the mediating variable is perceived control; and the control variables include gender, age, education background, individual privacy concerns, and perceived information overload.

We tested the participants’ privacy concerns and perceived information overload as two control variables besides demographics in this conceptual model. The existence of the effect of privacy concerns on the users’ privacy behaviors is still controversial because many studies have found a phenomenon privacy paradox wherein privacy concerns did not affect the users’ privacy behaviors, while many other studies posited that privacy concerns did affect the users’ privacy behaviors [62,63,64]. Perceived information overload is also regarded as a factor that could cause user privacy fatigue, leading to privacy behaviors [65].

Our aim is to test whether the presentation times of shared low-sensitivity information affect participants’ privacy behaviors irrespective of their levels of privacy concern. We also refer to a prior study [66] to control perceived information overload in the materials and procedures below. To enhance the validation of the conceptual model at the statistical level, we used the participants’ privacy concerns and perceived information overload as control variables.

## 3. Materials and Methods

To assess the conceptual model shown in Figure 1, we adopted a questionnaire survey experimental method [17,40] and created one pre-study and two online questionnaire surveys. The purpose of the pre-study was to test whether the participants could significantly perceive the different presentation times of shared low-sensitivity information (delayed or immediate) while eliminating the interference of information overload and other factors in the experimental materials used in the main studies. Study 1 tested the main and mediating effects of the model, and Study 2 tested the moderating effect of the model.

### 3.1. Pre-Study

To simulate real app browsing scenarios and exclude as much as possible the different preferences for online products, an e-book that ranked among the top ten most popular online shopping digital products according to the Ministry of Commerce of China was used as the product that was presented to the participants in the form of pictures in the pre-study. Participants’ trust and preference for different platforms and product brands were controlled by using “X” and “Y” as the names of the two apps, and “B” was used as the name of the product brand.

The pre-study featured two different presentation time conditions [40]. In the immediate condition, participants received a recommendation message based on the content of shared target information immediately when switching from app X to app Y. In the delayed condition, the participants received a recommendation message based on the content of shared target information after switching from app X to app Y and browsing three irrelevant content messages. The impact of information overload was controlled by setting the number of words as well as the speed and time of three irrelevant recommendation messages, referring to Liu’s study on non-information overload conditions [66].

The participants were randomly chosen to read the following notes: “Please imagine and put it into the following scenario: your mobile phone has installed apps X and Y at the same time, and you have already agreed to the users’ privacy policies of both apps and have begun to use them. One morning you opened your mobile phone and did the following things: first, clicked on app ‘X’ to view the configuration, price, and other information of brand B e-book”. The following message was then conveyed to the participants in the immediate condition: “Then, you opened another app ‘Y’ and saw the recommended information of brand B e-book”. On the other hand, the following message was sent to the participants in the delayed condition: “Then, you opened app ‘Y’ to browse the following information (three irrelevant content messages that control the time and the number of words), after which you saw the recommended information of brand B e-book.”

From 10 to 13 June 2022, we recruited 75 college students from a university in Fujian Province, China, including 39 males and 36 females (mean age = 25.4, SD = 3.64), to participate in the pre-study. The participants read two kinds of material carefully and completed the manipulation test items [67] for information presentation times in the two conditions (1 = low, 7 = high). The paired sample t-tests of the presentation times manipulation test items revealed the following: M_immediate_ = 3.468, M_delayed_ = 5.021 (*p* = 0.0007 < 0.001). There was a significant difference between the two groups in terms of the presentation time, indicating that the two types of material can be used as experimental manipulation materials.

### 3.2. Study 1

One of the main objectives of Study 1 was to investigate the effect of the information presentation time (immediate vs. delayed) on users’ privacy-regulation behavior and the mediating effect of online vigilance, thus testing the hypotheses H1–H3.

#### 3.2.1. Materials and Procedures

(1) Materials

The questionnaire comprised four parts. The first part presented the tested experimental stimulus materials in the pre-study. In the second part, the participants completed the control variables related to participants’ privacy concerns and information overload. In the third part, the participants finished the target information recognition item, online vigilance scale, and privacy-regulation behavior scale. The fourth part comprised the control variables related to participants’ gender, age, and educational background [17]. To the greatest extent possible, we adapted the constructs from the measurement scales used in prior studies to fit the context. A seven-point Likert scale was used for all the measurement scale items, with responses ranging from “strongly disagree” (1) to “strongly agree” (7). The online vigilance scale was adapted from Liang and Xue [28,29] and included three items. Privacy-regulation behavior was measured using Wolf’s scale [68] and included seven items across two subscales measuring the corrective and preventive behaviors. The privacy concern scale was adapted from the Internet users’ information privacy concerns scale [69]. The information overload scale was adapted from the perceived information overload scale from Chung et al. [70]. The full list of all items is illustrated in the Supplementary Materials.

To validate the questionnaire, we employed the content and face validity methods used in previous studies [71]. The questionnaire was first evaluated by four experts from the Department of User Behavior of E-commerce [72]. It was then read by 30 volunteers who were recruited from 2 universities in Fujian province, China, who were asked to provide any recommendations to ensure that every item was clear and readable. We then revised the questionnaire considering all the feedback and performed the survey using the online survey platform Credamo, which is one of the most commonly used survey platforms in China.

The unit of analysis was an independent user of the apps. The volunteer participant app users were recruited using Credamo online randomly to minimize any potential bias [73]. Each participant provided informed consent in order to be included in the study. The participants were then randomly sent the links to the two types of surveys previously tested in the pre-study and were rewarded with 3–5 RMB if they read the experimental scenario carefully and completed the test honestly and seriously. The application to conduct the studies was reviewed and approved by the Science and Technology Ethics Committee of Jimei University.

(2) Procedures

Participants (n = 220) aged 18–40 years were recruited voluntarily through the platform Credamo. Those who failed to pass the attention test, finished too fast, failed the target information recognition test, or repeatedly provided the same answer were excluded (n = 40). The data from 180 participants, including 89 and 91 in, respectively, the immediate and delayed groups (see Table 1), were analyzed.

The experiment controlled the following factors. One, to control the influence of forgetting the initial presentation in the delayed group, the participants were asked to perform the target information recognition test [74]. Two, as mentioned above, we also tested the participants’ privacy concerns and perceived information overload as control variables before the main test. Third, to control the influence of demographics, the participants were required to report the corresponding information (gender, age, and education level) after completing the experiment. In addition, the participants were told to complete the questionnaire anonymously to ensure their privacy; thus, they were expected to answer as truthfully as possible.

#### 3.2.2. Results of Study 1

(1) Reliability and validity test

SPSS 23.0 was used to test the reliability and validity of items and scales. The Cronbach’s α of the items that measured time manipulation, privacy-regulation behavior, online vigilance, privacy concerns, and perceived information overload ranged from 0.804 to 0.904, indicating acceptable to excellent internal consistency. The average variance extracted (AVE) values of the above items and scales ranged from 0.606 to 0.695, meeting the requirements of convergence validity.

(2) Multicollinearity diagnosis

A multicollinearity diagnosis was conducted using variance inflation factor (VIF) and tolerance values. The VIF of the independent, mediating, and control variables ranged from 1.033 to 1.260, and the tolerance values ranged from 0.783 to 0.968, which meets the accepted requirements of VIF (<10) and tolerance values (>0.1) [75], indicating no multicollinearity problem.

(3) Hypothesis test

First, an independent sample t-test was conducted on the effective items of presentation time manipulation. We found M_delayed_ = 5.930 (SD = 0.785) and M_immediate_ = 5.258 (SD = 0.936) (t = 5.224, *p* = 0.001 < 0.01), which indicated that the operation of information presentation time difference was effective.

Second, the main effect of presentation time on privacy-regulation behavior was tested: M_delayed_ = 4.438 (SD = 1.151) and M_immediate_ = 5.319 (SD = 1.190) (t = 5.244, *p* = 4.9 × 10^−7^ < 0.001). The impact of immediate and delayed information presentation time on user privacy-regulation behavior significantly differed. Thus, H1 is valid.

**Figure 2:**
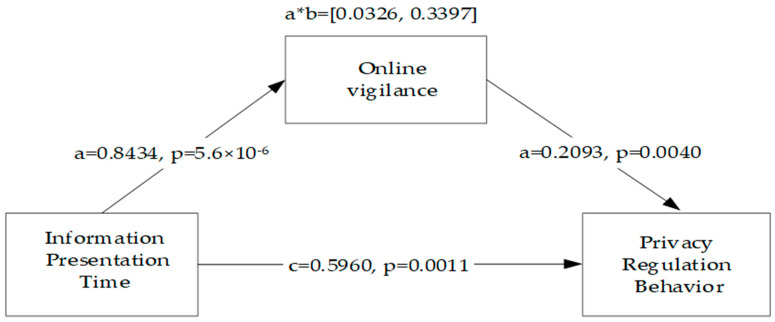
Mediating effect of online vigilance.

Third, we tested the mediating effect of online vigilance in the association between presentation time and privacy-regulation behavior. The independent sample t-test results showed that online vigilance differed significantly between the two groups M_delayed_ = 5.056 (SD = 1.275) and M_immediate_ = 6.036 (SD = 1.136); t = 5.447, *p* = 1.7 × 10^−7^ < 0.001. Then, we used the bootstrapping (5000 resamplings) method to test the mediating effect, as proposed by Hayes et al. (Model 4) [76]. The control variables were gender, age, educational background, perceived control, and privacy concerns. Figure 2 indicates the standardized coefficients and significance values for each path in the hypothesized model. According to the regression analysis results of the mediation model, presentation time had a significant positive impact on (1) online vigilance (β = 0.8434, *p* = 5.6 × 10^−6^) and (2) privacy-regulation behavior (β = 0.5960, *p* = 0.0011 < 0.01); online vigilance had a significant positive impact on privacy-regulation behavior (β = 0.2093, *p* = 0.0040 < 0.01); and education background had a significant positive impact on (1) online vigilance (β = 0.3848, *p* = 0.0003 < 0.001) and (2) privacy-regulation behavior (β = 0.3388, *p* = 0.0011 < 0.01). On the other hand, gender, age, educational background, and privacy concern level did not have significant impacts on online vigilance and privacy-regulation behavior.

Table 2 shows that the mediating effect of online vigilance was significant. Therefore, the mediating effect of online vigilance is only partial, and H2 and H3 are supported.

### 3.3. Study 2: The Regulatory Role of Perceived Control

#### 3.3.1. Materials and Procedures

Study 2 aimed to verify the robustness of the concept model and explore the moderating effect of perceived control. We adopted a 2 (information presentation time: immediate or delayed) × 2 (perceived control: high or low) two-factor inter-group design. Perceived control was measured and grouped by using the scores of the Perceived Control Scale adapted from Xu et al. [77]. The Cronbach’s α of the items was 0.78, indicating acceptable to excellent internal consistency, and the other measurement items were the same as in Study 1.

A total of 240 participants aged 18–40 years were recruited through the same online survey platform as in Study 1. The experimental process was the same as described for Study 1. The only change was that the target product was a kettle (instead of an e-book) to test the conceptual model’s robustness in different product categories. Finally, 199 informative participants were obtained, including 99 and 100 in, respectively, the immediate and delayed groups (see Table 3).

#### 3.3.2. Results of Study 2

(1) Reliability and validity test

SPSS 23.0 was used to test the reliability and validity of each scale and item. The Cronbach’s α of the measures of time manipulation, corrective behavior, preventive behavior, online vigilance, perceived control, and privacy concern ranged from 0.739 to 0.904, indicating acceptable to excellent internal consistency. The AVE value of each item and the scale ranged from 0.537 to 0.767, meeting the requirements of convergence validity.

The Cronbach’s α of the items that measured time manipulation, corrective behavior, preventive behavior, online vigilance, privacy concerns, perceived control, and perceived information overload ranged from 0.817 to 0.931, indicating acceptable to excellent internal consistency. The average variance extracted (AVE) values of the above items and scales ranged from 0.603 to 0.773, meeting the requirements of convergence validity.

(2) Multicollinearity diagnosis

The VIF of the independent variable, mediating variable, moderating variable, and control variables ranged from 1.060 to 1.353, and the tolerance value ranged from 0.739 to 0.940, which meets the accepted requirements of VIF (<10) and tolerance value (>0.1) [75], indicating no multicollinearity problem.

(3) Hypothesis test

First, an independent sample t-test was performed to test the effect of the presentation time. The results showed a significant difference between the two groups (M_delayed_ = 5.640 (SD = 0.857) and M_immediate_ = 5.283 (SD = 0.785); t = 3.058, *p* = 0.0031 < 0.01).

Second, we tested the moderating effect of perceived control on information presentation time and corrective and preventive behaviors, as well as the moderating effect of perceived control on online vigilance mediation. Bootstrapping (5000 resamples) was used to analyze the mediating effect of regulation (Model 15 in Hayes et al. [76]) (see Table 4).

Table 4 shows that educational background had a significant positive impact on CRB (β = 0.3723, *p* = 0.0003 < 0.001), while other control variables had no significant impact on CRB. The interaction items of PRT and PRC had a significant negative impact on CRB (β = −0.2519, *p* = 0.0403 < 0.05). PRC weakened the relationship between PRT and CRB—participants with low PRC had higher CRB under immediate conditions. The interaction of OV and PRC had a significant negative impact on CRB (β = −0.0912, *p* = 0.0048 < 0.05), which means that perceived control weakened the relationship between OV and CRB; that is, people with low PRC under high OV had higher CRB. Education had a significant positive impact on CRB (β = 0.3723, *p* = 0.0003 < 0.001), while other control variables had no significant impact on CRB.

**Figure 3:**
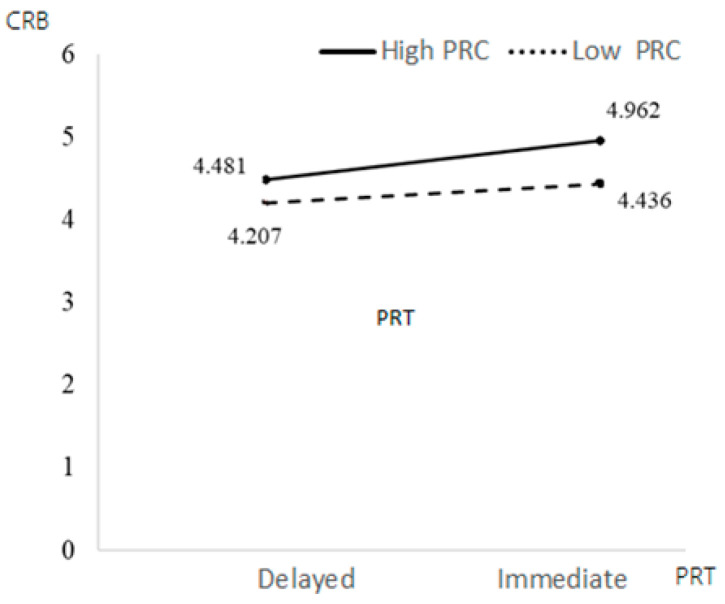
The moderating effects of PRT and PRC on CRB.

**Figure 4:**
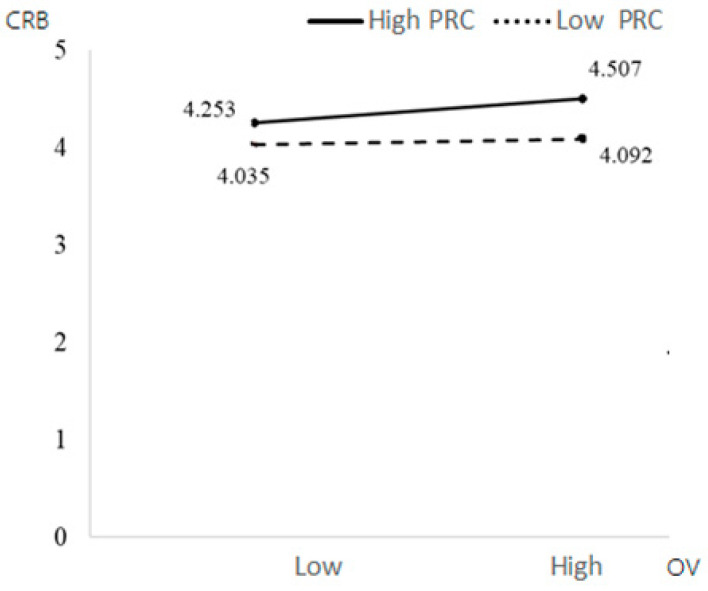
The moderating effects of OV and PRC on CRB.

**Figure 5:**
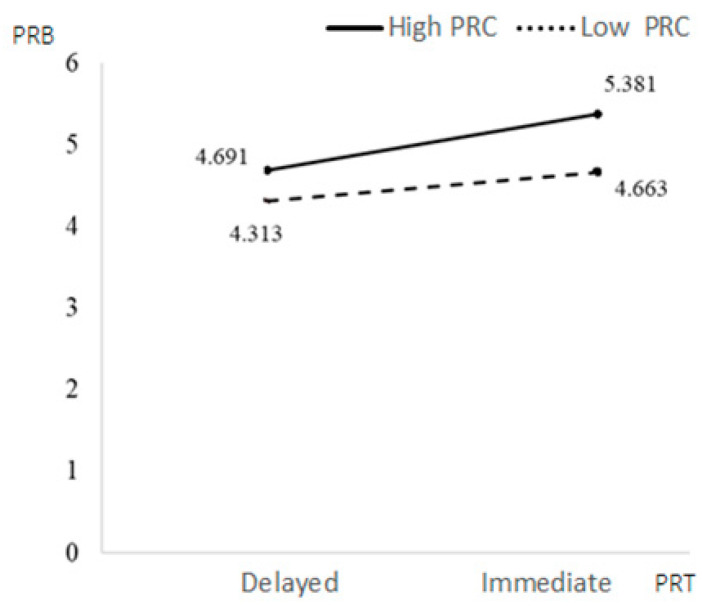
The moderating effects of PRT and PRC on PRB.

**Figure 6:**
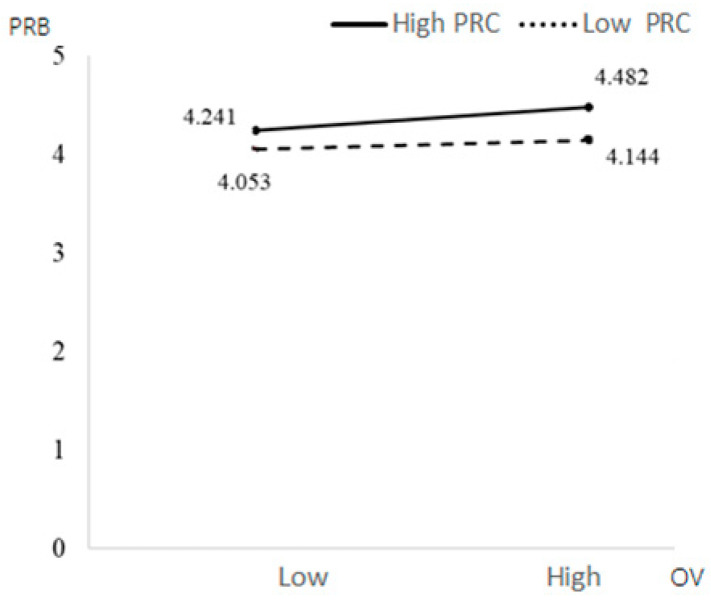
The moderating effects of OV and PRC on PRB.

Education background had a significant positive impact on PRB (β = 0.3296, *p* = 0.0028 < 0.05), while other control variables had no significant impact on PRB. The interaction term of the PRT and PRC had a significant negative impact on PRB (β = −0.3401, *p* = 0.0101 < 0.05). PRC weakened the relationship between PRT and PRB. Under the condition of immediate PRT, people with low PRC had higher PRB. The interaction of OV and PRC had a significant negative impact on PRB (β = −0.0882, *p* = 0.0109 < 0.05). PRC weakened the relationship between PRT and PRB; people with low PRC had higher PRB under the high OV condition. Education had a significant positive impact on PRB (β = 0.3723, *p* = 0.0003 < 0.001), while other control variables had no significant impact on PRB (see Figure 3, Figure 4, Figure 5 and Figure 6).

High and low perceived control groups were generated from the participants whose levels of perceived control were outside of the ± 1 SD range from the mean. According to the results of the moderated mediation effect on CRB (Table 5), the interaction of OV and PRC had a significant positive impact on CRB. The decision index was −0.0581, and the corresponding confidence interval was [−0.1402, −0.0065], indicating a valid moderated mediation effect. The negative value indicated that low PRC strengthened the mediating role of OV in the relationship between PRT and CRB, thereby enhancing CRB. The mediating effect value of OV under low perceived control was 0.2526, which was higher than the mediating effect value of 0.0704 under high perceived control. Individuals with low PRC demonstrated a stronger effect on CRB through OV.

The results of the test that measured the moderated mediating effect showed that PRC moderated the mediating effect of OV between PRT and PRB. The decision index was −0.0562, and the corresponding confidence interval was [−0.1435, −0.0063], indicating a true negative moderated mediating effect. This demonstrated that PRC weakened the mediating effect of OV between PRT and PRB. High PRC weakened the mediating role of OV in the relationship between PRT and PRB, thereby reducing PRB. The mediating effect value of OV under a low PRC was 0.2417, higher than that under a high PRC of 0.0656. Individuals with low perceived control had a stronger effect on PRB through OV. Therefore, H4 and H5 are supported.

Except for the pre-study, the two studies had data from 379 participants for final analysis, larger than the minimum sample size (250 responses) recommended by the multiple regression model [75,78].

## 4. Discussion

### 4.1. Theoretical Implications

This study aimed to explore whether the combination of cross-platform data flow and real-time recommendation algorithm technologies in vertical privacy contexts of multi-platforms communication changed users’ perceptions toward using low-sensitivity personal data on mobile multi-platform exchanges (i.e., vertical privacy context). The main conclusion is that the shared information presentation time between platforms affected users’ privacy-regulation behaviors. This finding aligned with the previous results based on CPM theory [79,80]. However, several differences exist between this work and prior studies. First, previous studies showed that the notification used by enterprises to publicly inform customers regarding data collection is a critical factor for customers’ willingness to use the recommended information and obviating their privacy behaviors [17,23,81,82]. Our study, based on PPM, showed that privacy-related behaviors could occur after the users have been informed about the data collection rules and even after they authorized sharing a part of their data at the beginning of using the apps via the corresponding privacy policies. Second, prior studies demonstrated that users only showed privacy reactions to the use of sensitive information [35,83,84]; however, our study proved that sharing and using low-sensitivity information also caused privacy-regulation behavior in the context of real-time recommendation technology and sharing data flow mode between platforms. This study confirmed the relationship between the presentation time of the recommended content generated by the low-sensitivity shared information between platforms and the users’ privacy-regulation behavior, thus expanding the privacy context characteristics of PPM [44]. Furthermore, we proved that the privacy concern, tested as a control variable, did not affect the participants’ privacy behaviors within the tested ranges of the presentation times of shared low-sensitivity information across platforms. This result is in line with the studies that suggested that the privacy paradox is caused by users’ delayed awareness or even total ignorance of the risks posed by the new big data technologies [65].

In terms of users’ perception of low-sensitivity information usage in real-time recommendations technology, this study verified the partial mediating role of online vigilance. Prior studies based on CPM and Technology Threat Avoidance theories showed that the collection and usage of personal sensitive information cause the perception of severe and sensitive privacy risks, thus leading to privacy behaviors [28,29]. However, these theories fail to explain why low-sensitivity information sharing, regarded as having low severity and sensitivity levels, could also lead to privacy behaviors [11]. This study showed that highly responsive instant technology could cause online vigilance perception of low-sensitivity information [45,46]. We verified that online vigilance played an important mediating role between the use and presentation time of shared information and privacy-regulation behavior, thus expanding the application of PPM and extending the mediating mechanism of the impact of information presentation time on user information behavior.

Finally, this study verified that a user’s perceived control affected that person’s strategies of privacy-regulation behavior caused by presentation time and online vigilance. Previous studies emphasized sensitive information usage and found that precautionary behavior was the most common response to the users’ privacy-regulation behavior [35,83,84]. This study found that low-sensitivity information could lead to both corrective and preventive regulation behavior. Perceived control moderated the main effect of information presentation time on privacy-regulation behavior and mediated the effect of online vigilance on privacy-regulation behavior. This result expands the understanding of the boundary conditions of the impact of information presentation time on privacy-regulation behavior.

### 4.2. Practical Implications

From the macro level of the policy system and the meso level of platform enterprises, studies have demonstrated that the sharing and flow of non- or low-sensitivity data between platforms are conducive to improving the utilization rate of data resources and creating greater value [85,86,87]. Although, at the vertical level, many online companies are investing substantially in cybersecurity, and a large amount of research on recommendation technology is also striving to desensitize and de-identify sensitive data for applications [86,87,88], from the micro perspective of user perception, the combination of information sharing between platforms and real-time recommendation technology is a “double-edged sword”. On the one hand, it will improve the pertinence and responsiveness of recommendation content; on the other hand, it will cause users to be vigilant and display privacy-related behavior regarding the collection and sharing of personal information across platforms.

In order to reduce users’ privacy-regulation behavior caused by the presentation time of low-sensitivity information sharing between platforms, this study suggests the following. First, there is a need to adjust the algorithm combination of information sharing and real-time recommendation to avoid using new, short-term, and immediate preferences across platforms to reduce the users’ online vigilance. Instead, we suggest focusing on analyzing users’ long-term and stable preferences regarding the shared information.

Second, from the perspective of reducing privacy behavior, digital platforms can regularly offer users information-protection measures options on the website, adopt more intuitive explanation flow charts to improve users’ information-protection skills, provide more channels to express users’ opinions, and encourage users to participate in the feedback improvement of recommendation algorithms. These measures will improve users’ perception of personal information control and reduce preventive behavior. Further, there is a need to make users aware of how platforms manage huge amounts of low-sensitivity data to reduce the negative impact of corrective behavior. For example, platforms can set up rules mandating the periodical removal of users’ low-sensitivity data.

### 4.3. Limitations

We note a few considerations that provide context for the conclusions that can be drawn from these findings. The first two limitations are directly related to the survey-based experimental setup. First, the times of information transfer and presentation tested in this work cannot fully represent the entire timeframe of users’ perceptions and reactions. Thus, additional time points can be used for longitudinal follow-up studies. Second, in the additional studies, EEG, eye movements, and other methods can be used to directly measure and verify user behavior. Third, the results of this study are directly applicable to 18–40-year-old adults; however, additional studies are required to test the applicability and robustness of the results to broader age groups and a larger number of participants. Finally, from the perspective of computer science, the effects of novel personalized recommendation algorithms and other features of information presentation generated by technological progress on user perception must be further explored.

## 5. Conclusions

By employing a survey experimental method based on PPM theory, this study showed that the information presentation time is a critical feature that would cause users to become vigilant and display privacy-regulation behaviors towards cross-platform sharing of low-sensitivity information in the vertical privacy context of multi-platform communication. Meanwhile, this study analyzed the mediating role of online vigilance and the moderating role of perceived control, expanding the scope of PPM and the understanding of users’ attitudes to utilizing low-sensitivity data. Therefore, reducing users’ negative perception and privacy-regulation behavior caused by the time factor of sharing information among platforms has important theoretical and practical value for the effectiveness of shared information flow across platforms and algorithm development and implementation. In the future, this work can serve as the basis for additional studies that explore other information presentation timeframes and additional categories of participants, such as those outside the tested age interval, as well as other features of information presentation to gain more insights into the complex interactions between human behavior and technological progress.

## Figures and Tables

**Table 1:** Participant profile in Study 1 (N = 180).

Variables		N	%
Gender	Male	78	43.3
	Female	102	56.7
Age (years)	Mean: 25.66		
	Range: 18–40		
	SD: 5.469		
Education level	Bachelor’s degree	114	63.3
	Master’s degree or above	38	21.1
	Junior college degree/other	28	15.6
Privacy concern	Low PC level (mean: 3.47, SD: 0.62)	98	54.4
	High PC level (mean: 5.27, SD: 0.66)	82	45.6
Information	Mean: 4.42		
Overload	SD: 1.11		

**Table 2:** Mediating effect of online vigilance.

	Effect	Standard Error	Lower Limit CI	Upper Limit CI
Indirect effect	0.1765	0.0790	0.0326	0.3397
Direct effect	0.5960	0.1803	0.2402	0.9518

Note: CI: confidence interval.

**Table 3:** Participant profile in Study 2 (N = 199).

Variables		N	%
Gender	Male	72	36.2
	Female	127	63.8
Age (years)	Mean: 25.97		
	Range: 18–40		
	SD: 5.529		
Education level	Bachelor’s degree	138	69.3
	Master’s degree or above	31	15.6
	Junior college degree/other	30	15.1
Privacy concern	Low PC level (mean: 2.86, SD: 0.75)	104	52.26
	High PC level (mean: 4.88, SD: 0.74)	95	47.74
Information Overload	Mean: 4.23 SD: 0.95		

**Table 4:** Moderating effect of perceived control.

	Corrective behavior				Preventive behavior			
	Coeff.	SE	t	*p*	Coeff.	SE	t	*p*
Constant	1.7593	0.8858	1.9862	0.0485	1.2437	0.9501	1.3090	0.1921
PRT	0.4811	0.1731	2.7788	0.0060	0.6906	0.1857	3.7186	0.0003
OV	0.2534	0.0587	4.3184	2.5 × 10^−5^	0.2411	0.0630	3.8300	0.0002
PRC	−0.0226	0.054	−0.4185	0.6760	−0.0381	0.0579	−0.6582	0.5112
PRT * PRC	−0.2519	0.1220	−2.0649	0.0403	−0.3401	0.1309	−2.5991	0.0101
OV * PRC	−0.0912	0.0320	−2.8526	0.0048	−0.0882	0.0343	−2.5707	0.0109
Gender	0.0247	0.1759	0.1405	0.8884	−0.0145	0.1887	−0.0771	0.9386
Age	0.0057	0.0157	0.3612	0.7183	0.0183	0.0168	1.0878	0.2781
Education	0.3723	0.1015	3.6690	0.0003	0.3296	0.1088	3.0285	0.0028
PC	0.0031	0.0693	0.0445	0.9646	−0.0386	0.0743	−0.5201	0.6036
IO	0.0264	0.0900	0.2930	0.7698	0.0042	0.0965	0.0436	0.9653
R^2^	0.3168				0.3307			
F	8.7171				9.2902			

Note: PRT: Presentation time; OV: Online vigilance; PRC: Perceived control; CRB: Corrective behavior; PRB: Preventive behavior; PC: Privacy concern; IO: Information overload.

**Table 5:** Analysis results of moderated mediating effects.

Moderating Variable	Mediating Variable	Dependent Variable	Mediating Effect				Mediated Effect			
			Effect Value	Standard Deviation	Lower Limit	Upper Limit	Determine Index	SD	Lower Limit	Upper Limit
Low PRC	OV	CRB	0.2526	0.0969	0.0797	0.4549	−0.0581	0.0347	−0.1402	−0.0065
High PRC			0.0704	0.0685	−0.0647	0.2166				
Low PRC	OV	PRB	0.2417	0.0860	0.0847	0.4168	−0.0562	0.0357	−0.1435	−0.0063
High PRC			0.0656	0.0787	−0.1116	0.2091				

## Data Availability

Data can be requested from the corresponding author.

## Supplementary Materials

The following supporting information can be downloaded at: https://www.mdpi.com/article/10.3390/bs13090706/s1, Table S1: Manipulation of Presentation Time; Table S2: Variables’ Measurements.
