# Supplementary material for: The Impact of Shared Information Presentation Time on Users’ Privacy-Regulation Behavior in the Context of Vertical Privacy: A Moderated Mediation Model
Source: Behav Sci (Basel). 2023 Aug 25;13(9):706. doi: 10.3390/bs13090706 (PMC10525935; doi:10.3390/bs13090706)
Supplement: Supplementary file 1 [file behavsci-13-00706-s001.zip › behavsci-2479798-supplementary.pdf]

Supplementary Materials

Table S1. Manipulation of Presentation Time

	Measurements	Source
Item 1	Rate the response speed of the recommendations provided by the app "Y".	Wang et al., 2022
Item 2	Rate how well the recommendations speed of the app "Y" fits your needs.	
Item 3	Rate the time required for the app "Y" to generate recommendations that fits your needs.	

Note: 7-point Likert scale:1 - very low, 7 - very high.

Table S2. Variables' Measurements

Variables			Measurements	Source
Perceived control		PRC 1	I believe l have control over who can get access to my personal information.	Xu et al., 2013 De Wolf et al., 2014
		PRC 2	I think I have control over what personal information is released and used by this website.	
		PRC 3	I believe I can control my personal information provided to this website.	
Online vigilance		OV 1	lt is likely that apps got authorized usage information across platforms.	Liang et al., 2014 Sharma et al., 2014
		OV 2	lt is likely that my browsing history on the app is monitored by other platforms.	
		OV 3	The content I was browsing through the app is being recommended quickly to me by other platforms.	
		OV 4	The app will quickly update information at least part of the time.	
Privacy regulation behavior	Preventive behavior	PRB 1	I will adjust privacy settings to limit the personal recommendations in the app "Y" .	De Wolf et al., 2014
		PRB 2	I will be careful with accepting authorized requests from third party services offering from across apps.	
		PRB 3	I will use different accounts to log in for different purposes.	
		PRB 4	I will make some customized visible group of friends in the app"Y"	
	Corrective behavior	CRB 1	I will delete my browsing history in the app "Y" .	
		CRB 2	I will cancel the authorization of the third party services which I used in app "Y".	
		CRB 3	I will delete my account and quit the app "Y" .	

Privacy Concerns	PC 1	It usually bothers me when companies ask me for personal information.	Malhotra et al., 2004
	PC 2	When companies ask me for personal information, I sometimes think twice before providing it.	
	PC 3	It bothers me to give personal information to so many companies.	
	PC 4	I am concerned that online companies are collecting too much personal information about me.	
lnformation Overload	IO 1	I could effectively handle every piece of information about the brand B e-book on this app.	Chung et al., 2023
	IO 2	There was too much information about the brand B e-book on this app that I was burdened by dealing with it.	
	IO 3	I feel overwhelmed with the number of recommendations I receive on this app about the brand B e-book.	

Note: 7-point Likert scale:1 - very disagree, 7 - very agree.
